# Postoperative results of ventilation tube insertion: a retrospective multicenter study for suggestion of grading system of otitis media with effusion

**DOI:** 10.1186/s12887-021-02855-1

**Published:** 2021-08-31

**Authors:** Chan Il Song, Byung Chul Kang, Chol Ho Shin, Yun Suk An, Tae Su Kim, Hyun Woo Lim, Hyun Joon Shim, Myung Hoon Yoo, Joong Ho Ahn

**Affiliations:** 1 Department of Otorhinolaryngology, Gangnam Severance Hospital, Yonsei University College of Medicine, Seoul, Korea; 2 Department of Otorhinolaryngology-Head and Neck Surgery, Ulsan University Hospital, University of Ulsan College of Medicine, Ulsan, Korea; 3 Department of Otorhinolaryngology-Head and Neck Surgery, Asan Medical Center, University of Ulsan College of Medicine, 88, Olympic-ro 43-gil, Songpa-gu, 05505 Seoul, Korea; 4 Department of Otorhinolaryngology-Head and Neck Surgery, Bundang Jesaeng General Hospital, Daejin Medical Center, Seongnam, Korea; 5 Department of Otolaryngology, School of Medicine, Kangwon National University, Chuncheon, Korea; 6 Department of Otolaryngology, Gangneung Asan Hospital, University of Ulsan College of Medicine, Gangneung, Korea; 7 Department of Otorhinolaryngology-Head and Neck Surgery, Eulji Medical Center, Eulji University School of Medicine, Seoul, Korea; 8 Department of Otorhinolaryngology-Head and Neck Surgery, School of Medicine, Kyungpook National University, Kyungpook National University Chilgok Hospital, Daegu, Korea

**Keywords:** Otitis media with effusion, Ventilating tube, Endoscopic otoscope, Grade of effusion

## Abstract

**Background:**

In otitis media with effusion (OME), it is important to know when to surgically intervene and when careful monitoring is more appropriate. This study aimed to visualize and classify the clinical manifestations of OME and the correlation between the new grading system and postoperative results after ventilation tube insertion (VTI).

**Methods:**

We classified the collective 1,012 ears from 506 patients into six groups: grade 0 (no effusion), grade I (scant effusion, but abnormal), grade II (effusion less than half of the tympanic cavity), grade III (effusion over half of the tympanic cavity, with air bubbles), grade IV (complete effusion), and grade V (retracted tympanic membrane or hemotympanum without air bubbles).

**Results:**

The mean age at VTI was 5.2 (±2.9) years and mean duration between diagnosis and operation was 4.1 (±1.8) months. Between the grades, the nature of the middle ear effusion was also significantly different (*p* < 0.001). The duration of ventilation tube retention after VTI was significantly different when compared between two groups: grade I-IV and grade V (*p* = 0.019). Our results showed that the recurrence rate, as well as rate of revision VTI, increased as the grade increased (*p* < 0.001).

**Conclusions:**

The new grading system of OME using endoscopic otoscope evaluation had a significant correlation with the age at VTI, the nature of middle ear effusion, the recurrence rate of OME, and the rate of revision VTI.

## Background

Otitis media with effusion (OME) is the most common ear disease in the pediatric population, and more than 60 % of all children have experienced otitis media at least once before the age of seven [1]. It is important to know when to surgically intervene and when careful monitoring is more appropriate. Medical treatments such as antihistamines, decongestants, and antibiotics are not recommended by clinical practice guidelines [2–5]. Ventilation tube insertion (VTI) is the preferred procedure when a child becomes a surgical candidate [4, 6]. However, it can be difficult to decide when to perform VTI surgery. There is also some discrepancy in clinical practice guidelines for OME management, which can vary by health-care system, time, and region [7].

Portable otoscope and pneumatic otoscope are the basic tools that physicians currently use to examine a patient’s tympanic membrane (TM). However, they have intrinsic limitations as illumination may not be optimal and the field of view is narrow. Endoscopic otoscopes, which use technology of digital acquisition and image storage, can be used by not only otorhinolaryngologists, but also other physicians after some basic training. They provide reliable medical photos that make it possible to compare the status of OME between patients or OME development over time in the same patient.

In a study, the prognosis of OME after VTI surgery was associated with the clinical manifestation such as the age at the time of VTI, the concurrent adenoidectomy, duration of ventilation tube retention, postoperative otorrhea, and early extrusion of the ventilation tube [8]. However, if the prognosis of OME can be predicted through endoscopic otoscope examination, it will help physicians to make the best decisions regarding treatment. So, we proposed a new grading system based on endoscopic otoscope evaluation in this study and tried to verify that it can provide reliable clinical results. The objectives of this study were to visualize and classify the clinical manifestations of OME and the correlation between the new grading system and postoperative results after VTI.

## Methods

### Subjects

We retrospectively evaluated 506 patients (321 males and 185 females) below the age of 15 years with unilateral or bilateral OME. Patients were diagnosed with OME after otoscopic examination, tympanometry, or pure tone audiometry, and only if they had no sign of acute inflammation including otalgia, fever, or perforation of the TM. Patients with recurrent acute otitis media were excluded because the purpose of this study was to evaluate the clinical manifestations and the correlation between the new grading system and postoperative results after VTI in only OME patients.

All of the patients underwent unilateral or bilateral VTI surgery in eight hospitals in Korea between January 2015 and December 2017, and otoscopic photos of the TMs before and after VTI surgery were taken with either a 2.7 mm or 4 mm telescope.

According to the American Academy of Otolaryngology–Head and Neck Surgery (AAO-HNS) guidelines [2] and the Korean clinical practice guideline: otitis media in children [4], patients with OME which has persisted longer than 3 months were considered subjects to VTI. The decision regarding VTI was made based on the bilaterality of the disease, duration of disease in the affected ears, hearing status, parental preference, and effects on developmental status of the child.

In all VTI surgeries, Paparella type tubes were used. After VTI surgery, we continued patient follow-up for at least 12 months. Patients with developmental delay, Down syndrome, cleft palate, or craniofacial disorders were excluded from this study.

### Grading system

**Fig. 1:**
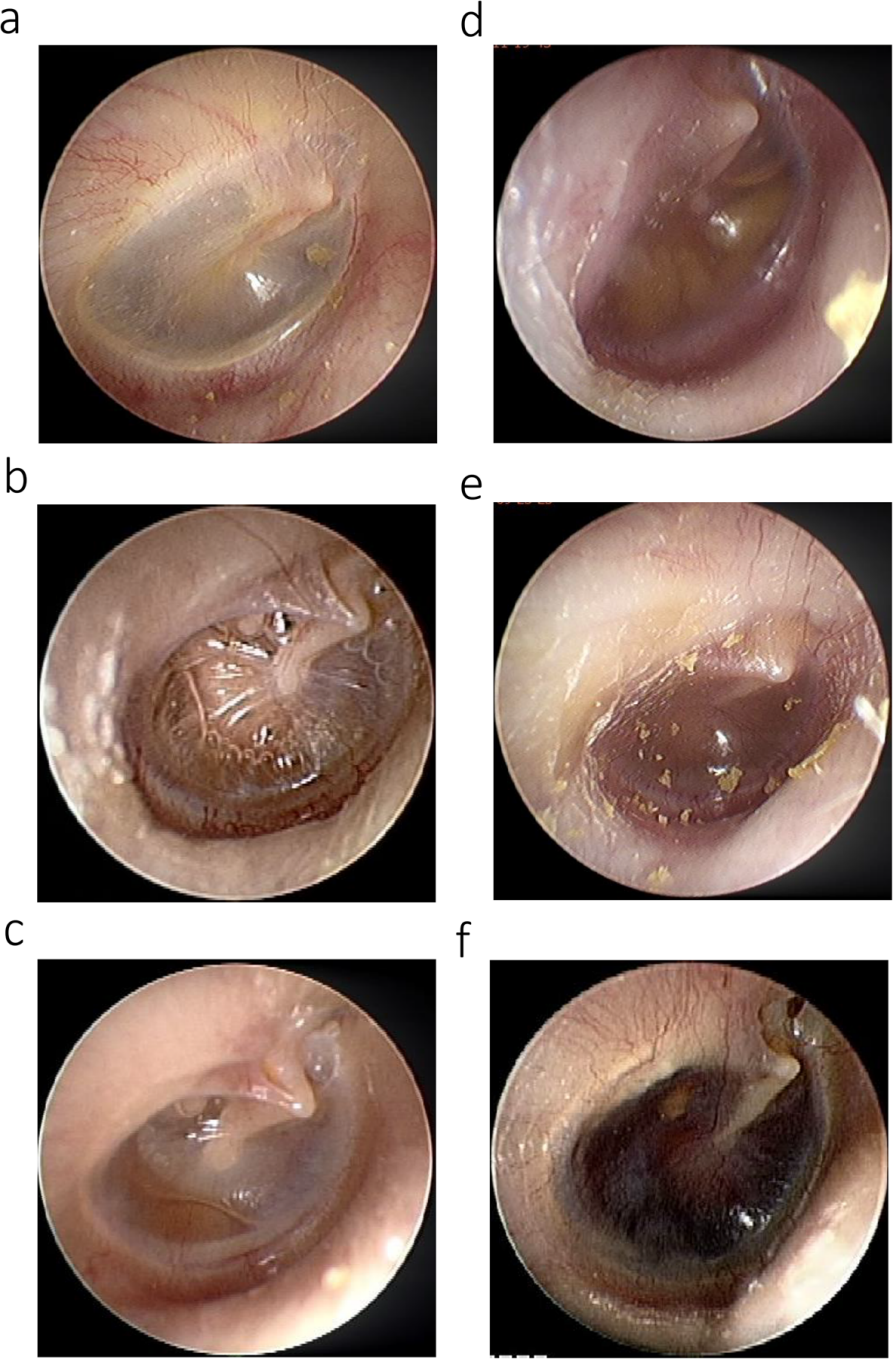
Criteria for the proposed otitis media with effusion grading system. **a**: Grade 0 is the normal condition without effusion. **b**: Grade I is scant effusion but abnormal tympanic membrane (TM). **c**: Grade II is effusion less than half of the tympanic cavity. **d**: Grade III is effusion over half of the tympanic cavity with air bubbles present. **e**: Grade IV shows effusion that is completely filling the middle ear cavity without bubbles. **f**: Grade V is retracted TM or hemotympanum

We classified the preoperative otoscopic photos from 1,012 ears into six groups: grade 0 (no effusion), grade I (scant effusion, but abnormal), grade II (effusion less than half of the tympanic cavity), grade III (effusion over half of tympanic cavity with air bubbles), grade IV (complete effusion), and grade V (retracted TM or hemotympanum without air bubbles) (Fig. 1). Tympanic membrane findings were first screened and classified by eight otologists and two experienced otologists then classified the otoscopic photos into 6 groups.

### Analyses

The nature of the middle ear effusion (MEE) after VTI surgery, duration of ventilation tube retention, recurrence of OME, and revision VTI surgery were analyzed according to the preoperative otoscopic findings and criteria for OME grading system that we proposed. Glue ear and mucoid otitis media as synonyms and the terminology was used according to the definition by Paparella [9].

### Statistical analyses

Statistical comparisons between different grades were carried out using the Chi-square test, one-way ANOVA test, Student’s t-test, and linear-by-linear association (SPSS software for Windows ver. 22, IBM, Armonk, NY, USA). p-values of less than 0.05 were considered to indicate a statistically significant difference.

### Ethical consideration

This retrospective study was approved for data collection and analysis by the Institutional Review Board of Severance Hospital, and informed consent was waived (IRB No. 3-2019-0067). All methods were carried out in accordance with relevant guidelines and regulations.

## Results

The mean age at VTI surgery was 5.3 (± 2.9) years. From group I to V, age at VTI was significantly different (*p* = 0.046). The mean duration between diagnosis and operation was 4.1 (± 1.8) months and the mean follow-up period after VTI was 24.2 (± 10.8) months. There was no significant difference in sex, laterality of OME, and preoperative OME duration (Table 1).

**Table 1:** Descriptive characteristics of the patients

		Grade 0 (*n* = 60)	Grade I (*n* = 32)	Grade II (*n* = 57)	Grade III (*n* = 152)	Grade IV (*n* = 410)	Grade V (*n* = 301)	*p-*value
Age (year)		7.0 ± 3.6^a^	6.3 ± 3.6	5.3 ± 3.1	5.1 ± 3.0	4.9 ± 2.3	5.5 ± 3.0	< 0.001^b^ 0.046^c^
Sex								0.591
Male		40	22	41	90	257	192	
Female		20	10	16	62	153	109	
Laterality								0.748
Right		27	16	33	80	200	150	
Left		33	16	24	72	210	151	
Preoperative OME duration (month)		-	3.6 ± 1.5	3.8 ± 2.0	3.9 ± 1.6	4.1 ± 1.9	4.1 ± 1.8	0.056

Note: Values are presented as mean ± standard deviation or number

*VTI* ventilation tube insertion, *OME* otitis media with effusion

^a^Age at VTI in the contralateral ear because ear with grade 0 did not undergo VTI

^b^*p* -value for difference in mean age from grade 0 to grade V

^c^*p* -value for difference in mean age from grade I to grade V

**Fig. 2:**
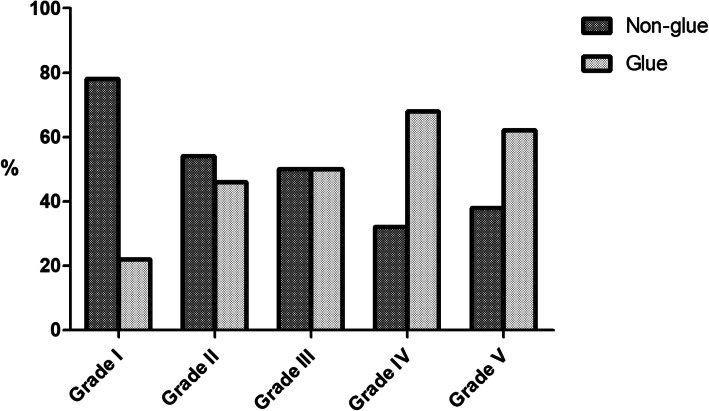
Comparison of the nature of the middle ear effusion. The ratio of non-glue to glue ear was 78% and 22% in grade I, 54% and 46% in grade II, 50% and 50% in grade III, 32% and 68% in grade IV, and 38% and 62% in grade V, respectively. Statistically, the higher the grading, the higher the ratio of glue ear (*p *< 0.001)

The nature of the MEE was categorized into glue, serous fluid, and no effusion after VTI surgery. Between the grades, nature of MEE was significantly different (*p* < 0.001). Of 32 ears with grade I classification, 17 (53 %) had no effusion, 8 (25 %) had serous fluid, and 7 (22 %) had glue. Fifty-seven ears were included in the grade II group. Seven ears (12 %) with grade II had no effusion, 24 (42 %) had serous fluid, and 26 (46 %) had glue. Of 152 ears with grade III classification, 13 (9 %) had no effusion and 63 (41 %) had serous fluid. Seventy-six ears (50 %) with grade III otoscopic findings had glue in the middle ear. Grade IV otoscopic findings were seen in 410 ears. Out of the 410, 16 (4 %) had no effusion, 115 (28 %) had serous fluid, and 279 (68 %) had glue. Of the 301 OME ears with grade V classification, 23 (8 %) had no effusion, 91 (30 %) had serous effusion, and 187 (62 %) had glue in middle ear cavity. In comparison, the nature of MEE was categorized into glue and non-glue effusion, and the higher the grading, the more ears had a glue effusion in middle ear cavity (*p* < 0.001; Fig. 2).

**Fig. 3:**
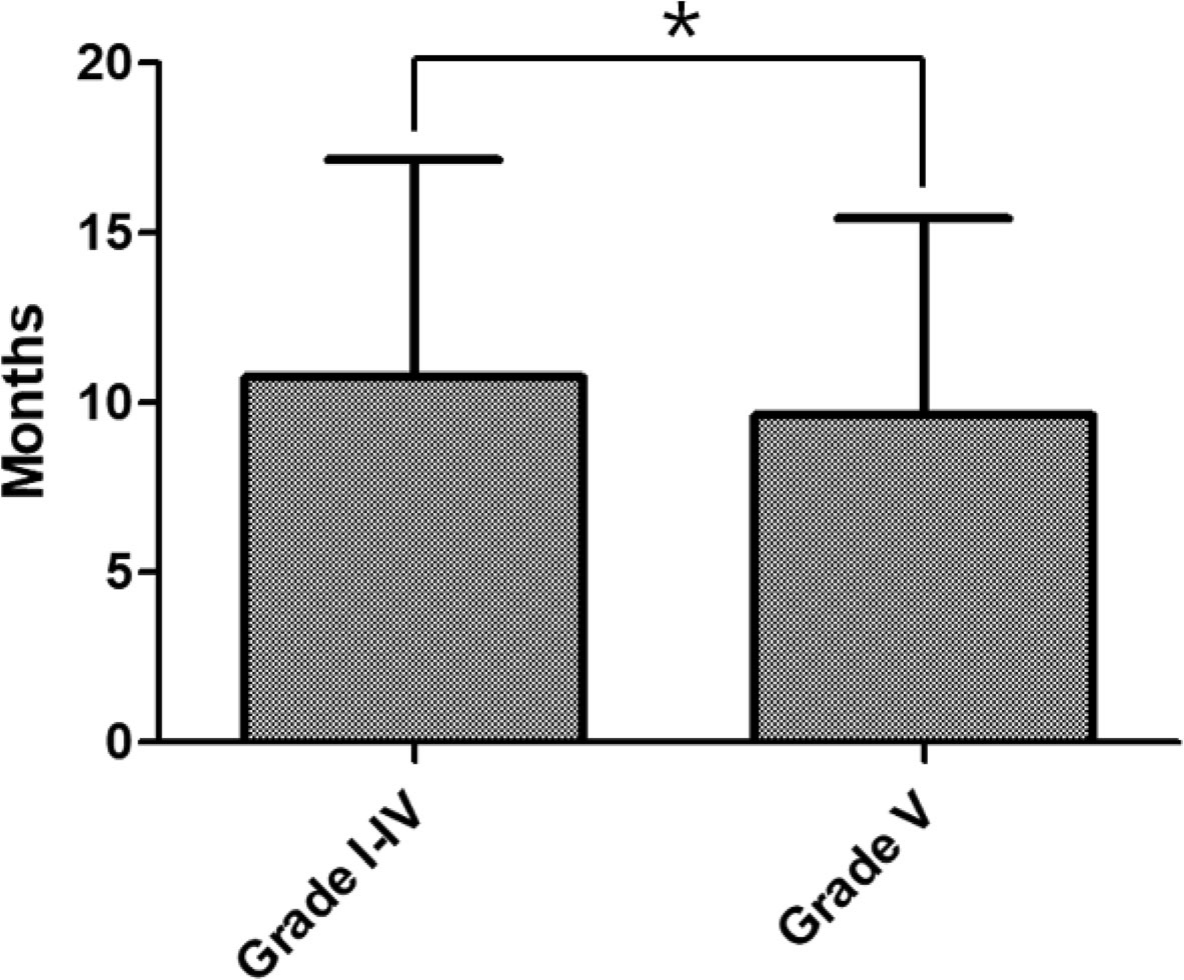
Comparison of ventilation tube retaining duration. Duration of ventilation tube retention after ventilation tube insertion surgery was significantly different when compared between two groups: grade I-IV was 10.8 (±6.4) and grade V was 9.6 (±5.8) (*p *= 0.019)

The mean duration of ventilation tube retention after VTI surgery was 10.3 months for all ears. Mean duration of ventilation tube retention of grade I was 9.9 (± 6.9) months. From grade II to V, mean duration was 9.8 (± 3.0), 10.7 (± 7.0), 10.9 (± 5.8), and 9.6 (± 5.8), respectively and it was not significantly different between grades (*p* = 0.226). Duration of ventilation tube retention after VTI surgery was significantly different when compared in two groups: grade I-IV and grade V (*p* = 0.019; Fig. 3).

**Fig. 4:**
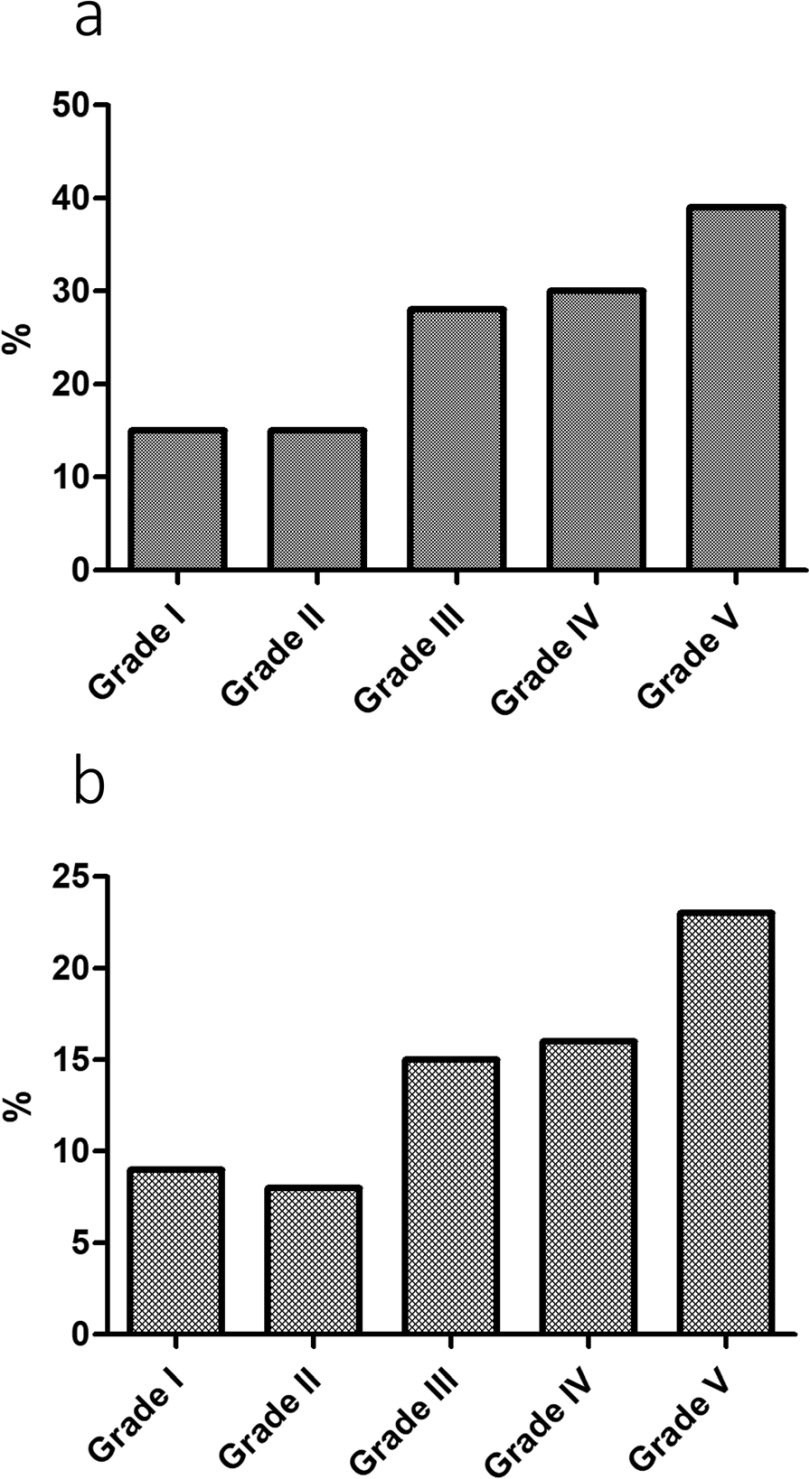
Prognosis according to the preoperative otitis media with effusion (OME) grading system. **a**: From grade I to grade V, recurrence rate of OME was 15.6%, 15.7%, 28.9%, 30.2%, and 39.5%, respectively. The recurrence rate increased as the grade increased (*p *< 0.001).**b**: The rate of revision ventilation tube insertion (VTI) surgery was 9.4% in grade I, 8.8% in grade II, 15.8% in grade III, 16.6% in grade IV, and 23.3% in grade V. Also, revision VTI surgery rate increased as the grade increased (*p *< 0.001)

From grade I to grade V, recurrence rate of OME during follow-up period was 15.6 %, 15.7 %, 28.9 %, 30.2 %, and 39.5 %, respectively. The recurrence rate increased as the grade increased (*p* < 0.001). Ear with grade 0 TM finding had no OME during follow-up period. The rate of revision VTI surgery was 9.4 % in grade I, 8.8 % in grade II, 15.8 % in grade III, 16.6 % in grade IV, and 23.3 % in grade V. Also, revision VTI surgery rate increased as the grade increased (*p* < 0.001; Fig. 4).

## Discussion

The present study indicated that the new grading system of OME had a significant correlation with the postoperative outcome of VTI as a treatment of OME. The treatment results of OME were already known through many studies, and long-term follow-up results have also been reported [10, 11]. However, studies on the endoscopic otoscope findings of OME are still insufficient.

Endoscopic otoscope findings of OME vary depending on when the inspections were performed. However, the most participants of this study had chronic and stable TM findings, because the mean preoperative OME duration was more than 3 months and patient with recurrent acute otitis media were excluded. In other words, the purpose of this study was to predict the prognosis of chronic OME with Endoscopic otoscope findings. So, patients with recurrent acute otitis media were excluded.

Audiological tests including tympanometry and pure tons audiometry were used for the diagnosis of OME. However, in many cases, patients were diagnosed with OME after only otoscopic examination, and it was impossible to analyze the results of hearing test statistically because of the small number. Before the VTI surgery, for patients without an age-appropriated hearing test, authors provided enough education families of patients with OME regarding the natural history of OME, need for follow-up, and the possible sequelae and obtained the consents for the VTI surgery.

All participants of this study underwent unilateral or bilateral VTI. The VTI surgeries were also performed for the ear with OME in patients with unilateral OME. However, in patients with unilateral OME, the tendency to wait longer before surgical intervention compared to those with bilateral OME. This might cause the difference in the age at ipsilateral or contralateral VTI.

As we expected, the nature of the MEE was getting worse from grade I to grade V: the higher the grading, the more ears had glue contents. It showed a statistically significant difference. Of 32 ears with grade I preoperatively, seventeen (53 %) had no middle ear effusion. On the other hand, only 59 (6.4 %) out of 920 ears with grade II, III, IV, and V showed no effusion in middle ear cavity during VTI surgery. Some researchers have reported the development of OME after surgery under general anesthesia [12–14]. In one study, MEE accumulated after surgery under general anesthesia in 3.3 % of participants [15]. However, mask ventilation during induction of general anesthesia was performed in some hospitals for surgeries with short operating times, and this procedure induced the positive pressure in the Eustachian tube and middle ear cavity. Sometimes, similar to our findings, this can improve the condition of the middle ear cavity and the there is no MEE noted during VTI surgery in patients with definite OME, such as those with grade I - V.

Glue ear has many pathological and clinical differences from OME with serous fluid including mucosal metaplasia, ciliary motility, and natural course [16]. A comparison of the ratio between non-glue ear and glue ear showed a statistically significant difference.

The mean duration of ventilation tube retention after VTI surgery was not significantly different between each grade. However, the duration of ventilation tube retention showed significant difference when compared between two groups: grade I - IV and grade V. Grade V is the condition with bleeding present or with morphological changes of the TM. This study showed that patients with grade V disease tend to have early ventilation tube extrusion, a high rate of recurrence, and a high rate of revision VTI surgery.

## Conclusions

A new grading system of OME using endoscopic otoscope was proposed and it showed a significant correlation with the age at VTI, the nature of MEE, the recurrence rate of OME, and the rate of revision VTI surgery. This new grading system of OME using endoscopic otoscope may help clinicians make better therapeutic plans, and patients to better understand their disease progression and likely outcomes.

## Data Availability

The datasets are available from the corresponding author on reasonable request.

## References

[1] Todberg T, Koch A, Andersson M, Olsen SF, Lous J, Homøe P (2014). Incidence of otitis media in a contemporary Danish National Birth Cohort. PLoS One.

[2] Rosenfeld RM, Shin JJ, Schwartz SR, Coggins R, Gagnon L, Hackell JM (2016). Clinical practice guideline: otitis media with effusion (update). Otolaryngol Head Neck Surg.

[3] Ito M, Takahashi H, Iino Y, Kojima H, Hashimoto S, Kamide Y (2017). Clinical practice guidelines for the diagnosis and management of otitis media with effusion (OME) in children in Japan, 2015. Auris Nasus Larynx.

[4] Lee HJ, Park SK, Choi KY, Park SE, Chun YM, Kim KS (2012). Korean clinical practice guidelines: otitis media in children. J Korean Med Sci.

[5] National Collaborating Centre for Women’s and Children’s Health (2008). National Institute for Health and Clinical Excellence: Guidance. Surgical management of otitis media with effusion in children.

[6] Rovers MM, Schilder AG, Zielhuis GA, Rosenfeld RM (2004). Otitis media. Lancet.

[7] Clay-Williams R, Stephens JH, Williams H, Hallahan A, Dalton C, Hibbert P (2020). Assessing the appropriateness of the management of otitis media in Australia: a population-based sample survey. J Paediatr Child Health.

[8] Ahn JH, Yoon TH, Pae KH, Kim TS, Chung JW, Lee KS (2006). Clinical manifestations and risk factors of children receiving triple ventilating tube insertions for treatment of recurrent otitis media with effusion. Pediatrics.

[9] Paparella MM (1976). Middle ear effusions: definitions and terminology. Ann Otol Rhinol Laryngol.

[10] Cayé-Thomasen P, Stangerup SE, Jørgensen G, Drozdziewic D, Bonding P, Tos M (2008). Myringotomy versus ventilation tubes in secretory otitis media: eardrum pathology, hearing, and eustachian tube function 25 years after treatment. Otol Neurotol.

[11] Khodaverdi M, Jørgensen G, Lange T, Stangerup SE, Drozdziewizc D, Tos M (2013). Hearing 25 years after surgical treatment of otitis media with effusion in early childhood. Int J Pediatr Otorhinolaryngol.

[12] Johnson LP, Parkin JL, Stevens MH, Otto WC, McCandless GA (1980). Action of general anesthesia on middle ear effusions. Arch Otolaryngol.

[13] Tom LW, Tsao F, Marsh RR, Kessler A, Konkle DF (1994). Effect of anesthetic gas on middle ear fluid. Laryngoscope.

[14] Koivunen P, Alho OP, Uhari M, Partanen A, Luotonen J (1996). General anesthesia with and without nitrous oxide (N2O) and the weight of middle ear effusion in children undergoing adenoidectomy and tympanostomy. Laryngoscope.

[15] Kanai R, Kaneko K (2012). Negative middle ear pressure and otitis media with effusion after surgery under general anesthesia. Acta Otolaryngol.

[16] Rinaldo A, Ferlito A (2000). The pathology and clinical features of “glue ear”: a review. Eur Arch Otorhinolaryngol.

